# Belatacept as an alternative immunosuppressive agent for bone marrow-sparing in idiopathic pulmonary fibrosis lung transplant recipients with short telomeres

**DOI:** 10.1016/j.healun.2025.04.022

**Published:** 2025-05-07

**Authors:** Stefanie J. Hannan, Carlo J. Iasella, Michel Sciullo, Cody Moore, Ryan Rivosecci, Lauren Sacha, Rachel M. Sutton, Ritchie Koshy, Norihisa Shigemura, Pablo G. Sanchez, Rafic Farah, Chadi A. Hage, Jonathan K. Alder, John F. McDyer

**Affiliations:** a Division of Pulmonary, Allergy, Critical Care and Sleep Medicine, University of Pittsburgh, Pittsburgh, Pennsylvania; b Department of Pharmacy and Therapeutics, University of Pittsburgh, Pittsburgh, Pennsylvania; c Medical Center, Hillman Cancer Center, University of Pittsburgh, Pittsburgh, Pennsylvania; d Lung Transplant Research Center, University of Pittsburgh, Pittsburgh, Pennsylvania; e Department of Cardiothoracic Surgery, University of Pittsburgh, Chicago, Illinois; f Department of Surgery, University of Chicago.

**Keywords:** pulmonary fibrosis, lung transplantation, telomere, belatacept, post-transplant, lymphoproliferative, disorder

## Abstract

As we have previously shown, idiopathic pulmonary fibrosis lung transplant recipients (IPF-LTRs) with short-telomere length are prone to develop significant cytopenias and poor tolerance to cell-cycle inhibitors, specifically mycophenolate mofetil (MMF), post transplant. We investigated the use of belatacept as an alternative immunosuppressive agent in a prospective, open-label cohort of 9 ST-IPF-LTRs at our institution. These patients were either challenged with MMF (majority) or immediately started on belatacept post transplant with the goal to bridge to everolimus, an mammalian target of rapamycin inhibitor that is commonly used post transplant. We describe outcomes in the first-year post transplant, including the incidence of acute cellular rejection, Epstein-Barr virus viremia, and 1 case of post-transplant lymphoproliferative disorder (PTLD) at 13 months. The use of belatacept postlung transplant may be an acceptable short-term alternative therapy to cell-cycle inhibitors in ST-IPF-LTRs with cytopenias but may lead to a higher risk of Epstein-Barr Virus viremia and PTLD when belatacept is used long term in these patients.

## Brief Communication

Our earlier work has demonstrated that the majority of idiopathic pulmonary fibrosis lung transplant recipients (IPF-LTRs) are enriched for lymphocyte short-telomere length (STL) (> 50%) and approximately 24% harbor germline mutations in the major telomere-maintenance genes: *TERT, TERC* (or *TR*)*, RTEL1, PARN, TINF2, DKC1*, and *NAF1*.^1–3^ We and others have shown that STL IPF-LTRs are prone to develop significant cytopenias post transplant with poor tolerance predominantly to cell-cycle inhibitors, such as mycophenolate mofetil (MMF), along with other bone marrow suppressive therapies.^1^ To this end, we investigated the use of early post-transplant belatacept as an alternative, bone marrow-sparing immunosuppressive agent to replace the use of a cell-cycle inhibitor therapy. Here, we report a pilot study of 9 STL IPF-LTRs (all double-lung transplants) who were successfully treated with early post-transplant belatacept at our institution.

**Figure 1:**
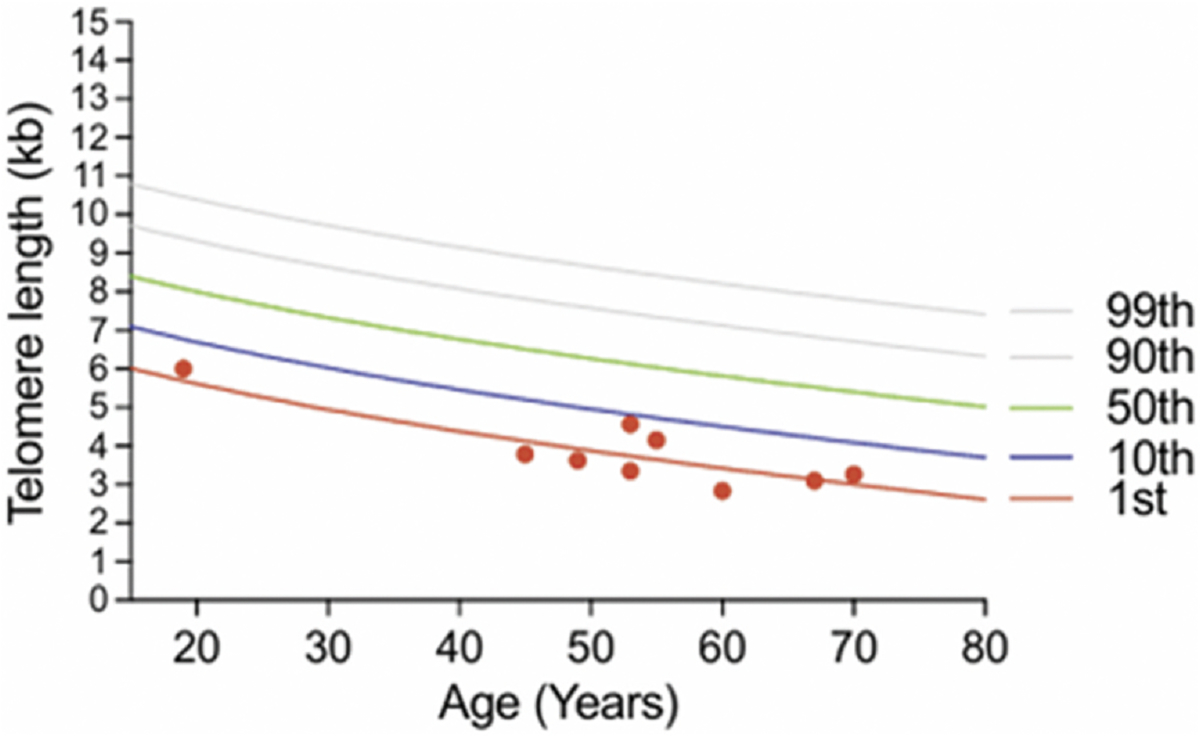
Lymphocyte telogram of the entire cohort. Lymphocyte telomere length was measured using FlowFISH on the short-telomere-idiopathic pulmonary fibrosis lung transplant recipients cohort (*n* = 9). This telogram shows that the entire cohort has short-telomere length < 10th percentile and 6/9 (67%) ≤1st percentile.

**Figure 2:**
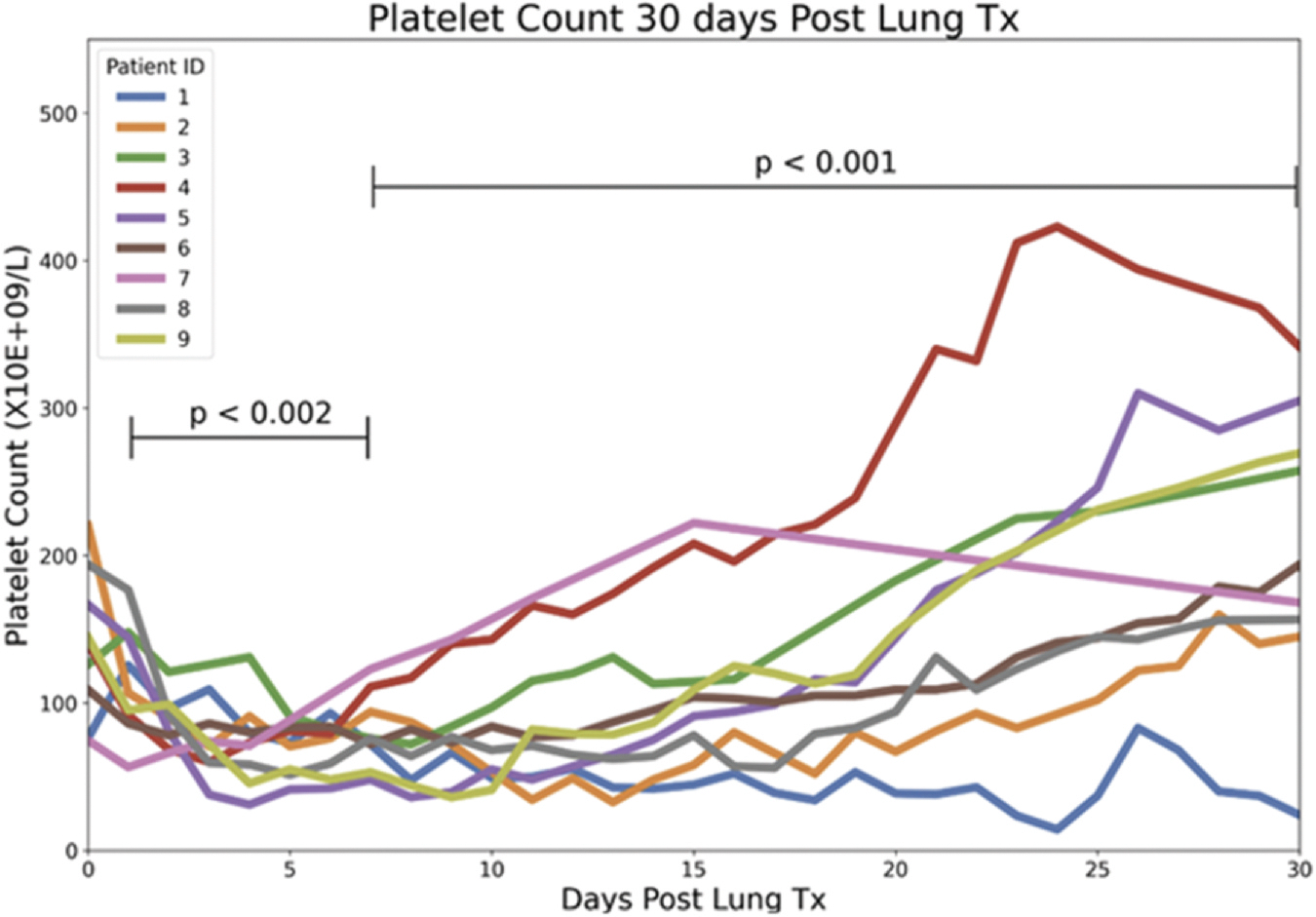
We observed a significant fall in platelet counts across the cohort from immediately post transplant to platelet nadir (*p* < 0.002), with a median of 7 days. Following the discontinuation of mycophenolate mofetil, platelet counts significantly increased across the cohort in 8/9 patients by day 30 (*p* < 0.001).

Our study of 9 STL IPF-LTRs who underwent double-lung transplant was conducted between 2018 and 2024, and the cohort, which is predominantly male (77%), as shown in Table 1. All patients had lymphocyte telomere length measured by flow cytometry and fluorescence in-situ hybridization (flowFISH), which revealed 4/9 (44%) had TL < 1st percentile, 2/9 (22%) at the 1st percentile, and 3/9 (33%) between the 1st and 10th percentile (Table 1; Figure 1). Additionally, granulocytes were measured in 8/9 with 7/8 (88%) < 1st percentile and 1/8 (11%) between the 1st and 10th percentile (data not shown). Further, 7/9 (77%) underwent genetic characterization using a targeted sequence panel for the telomere-maintenance genes, with 5/7 (71%) having a rare gene variant: 2 in *RTEL1*, 2 in *TERT*, and 1 in *TINF2*. Induction therapy consisted of Basiliximab in 8/9 (88%) and Thymoglobulin in 1 (patient #5). Notably, all 7/9 patients at risk for cytomegalovirus (CMV) received Letermovir prophylaxis instead of Valganciclovir for bone marrow-sparing. Importantly, 8/9 (88%) were challenged with MMF in the immediate post-transplant period, with a median discontinuation at 4 days. Patient #1 was not challenged with MMF due to myelodysplastic syndrome diagnosed prelung transplant. We observed a significant fall in platelet counts across the cohort from immediately post transplant to platelet nadir (*p* < 0.002) with median at 7 days (Figure 2). Following the discontinuation of MMF, platelet counts significantly increased across the cohort in 8/9 patients by day 30 (*p* < 0.001, Figure 2). Patient #1 had persistent thrombocytopenia in the context of fulminant bone marrow failure requiring regular platelet transfusions as a bridge to bone marrow transplant, 6 months postlung transplant. With the exception of patient #1, no other patients in our cohort required transfusions or had significant bleeding episodes during the immediate post-transplant period.

Our intention was to use belatacept therapy as a bridge to treatment with the mammalian target of rapamycin (mTOR) inhibitor, everolimus, by 3 months post transplant due to the previously reported association of mTOR inhibitor therapy increasing the risk for airway dehiscence in the early post-transplant period. We were successful in bridging belatacept to everolimus therapy in 6/9 (67%) patients in our cohort. However, 3 transitions were unsuccessful due to renal failure and/or recurrent cytopenias. The duration of belatacept therapy was a median of 3 months in the patients who transitioned to everolimus therapy and 10 months in those who did not transition, with 1 patient remaining on long-term belatacept at the time of this writing.

We also evaluated the incidence of acute cellular rejection (ACR) within the first-year postlung transplant. In our cohort, only 1/9 (11%), patient 8, developed ACR on day 288 post transplant. They received standard treatment with methylprednisolone and repeat transbronchial biopsy demonstrated resolution of rejection. There were no cases of antibody-mediated rejection or de novo donor-specific antibody identified within the cohort during the first-year post transplant.

A previous report of 52 LTRs receiving a single dose or maintenance belatacept described cases of CMV, Epstein-Barr virus (EBV) + post-transplant lymphoproliferative disorder (PTLD), and invasive fungal infections. However, this cohort was not compared to standard IS controls.^4^ Close monitoring for CMV and EBV was performed in all patients. We found that 2/9 (22%) developed CMV viremia and received treatment with control of viremia. Patient #1 was a CMV mismatch and developed viremia 16 months after prophylaxis was discontinued. Patient #5 was D−/R+ and developed viremia after prophylaxis was stopped at 6 months. Neither patient developed end-organ complications of CMV infection. We did not observe any cases of invasive fungal infection in our cohort within 1 year of receiving belatacept, and fungal prophylaxis was divided as follows: Isavuconazole *n* = 5, Voriconazole *n* = 3, Posaconazole *n* = 1. As shown in Table 1, all 9 patients were EBV seropositive. We noted 3/9 (33%) ST-IPF-LTRs developed EBV viremia, without PTLD, and were responsive to Rituximab therapy. Patient 8 developed EBV viremia during maintenance belatacept (stopped at month 11) and EBV-associated PTLD at 13 months. The patient is undergoing treatment with Rituximab at the time of this writing. Notably, 3/4 patients who developed EBV viremia or PTLD received long-term belatacept (range 8–20 months). In each case, prolonged belatacept treatment was due to chronic kidney disease precluding a transition to everolimus. Notably, patient #8, who developed PTLD, also had an episode of ACR at 10 months post transplant (before PTLD), highlighting the challenge of maintaining higher levels of immunosuppression that may result in complications. Interestingly, the overall incidence of ACR in this small cohort was unexpectedly low (1/9, 11%), as our previous study showed similar rates of ACR in ST-LTRs compared to controls (*n* = 149).^5^ This suggests a possible increased level of immunosuppression with the addition of belatacept, which is known to disrupt T-cell costimulatory signals. Our close monitoring of EBV viral loads in these patients was based on our earlier report that showed patients with IPF were at increased risk for PTLD.^6^ In that study, we did not measure telomere length but subsequently showed that the majority of IPF-LTRs had short-TL.^5^ Belatacept therapy was first explored in kidney transplant, and the most recent study in kidney transplant recipients from the UNOS database found that, while the risk for PTLD was higher in patients on belatacept therapy compared to CNI-based immunosuppression, this risk remained low after adjusting for differences in patient characteristics.^7^ However, data on this topic in LTRs are more limited. Our prior report on a small cohort of LTRs treated with belatacept at our center did not observe patients developing PTLD.^8,9^ More recently, an RCT by Huang et al^10,11^ evaluated belatacept-based therapy versus standard IS, where CNI was removed after 90 days. Unfortunately, this study had to be prematurely stopped due to a high mortality rate in the belatacept cohort 3/13 (23%) during the trial. However, another 3 died after the trial was discontinued for a total of 6/13 (46%). While 2 of these deaths were due to COVID-19 infection, another 2 were due to vascular complications, 1 patient developed postviral RAS, and another patient EBV + PTLD.^10,11^ This study significantly differed from the current study in that CNI therapy was maintained and belatacept substituted for cell-cycle inhibitor therapy. However, a case of PTLD was common to both studies at a combined risk of 2/22 (9%), which is higher than the reported average incidence in LTRs.^6^ The 1 death observed in the current study 1/9 (11%) was due to pneumonia in addition to CLAD and failure to thrive.

Collectively, given the increased risk of PTLD in IPF-LTRs^6^ and the susceptibility of STL IPF-LTRs to herpesvirus infections,^12,13^ caution is warranted particularly in extended duration of belatacept therapy in LTRs given our findings. In conclusion, we find that belatacept therapy may be an acceptable alternative immunosuppressant in STL IPF-LTRs who do not tolerate cell-cycle inhibitors due to significant cytopenias. However, more studies are needed to define the risks and potential uses of belatacept therapy in LTRs.

## Figures and Tables

**Table 1:** Baseline Characteristics of Study Cohort

ID #	Gender	Age at transplant	Lymphocyte TL (percentile)	Mutant telomere gene	MMF Trial	Days on MMF post-LTx	Length of belatacept (months)	ACR	EBV viremia	PTLD

1	M	20	1st	*RTEL1*	No	0	3	-	-	-
2	M	45	< 1st	*TINF2*	Yes	5	3	-	-	-
3	M	54	1st-10th	None	Yes	11	3	-	-	-
4	F	61	< 1st	*TERT*	Yes	3	13	-	-	-
5	F	57	1st-10th	*TERT*	Yes	2	8	-	+	-
6	M	70	1st-10th	Not done	Yes	1	20	-	-	-
7	M	49	< 1st	None	Yes	4	19	-	+	-
8	M	53	< 1st	*RTEL1*	Yes	3	10	+	+	+
9	M	68	1st	None	Yes	7	3	-	+	-

Abbreviations: ACR, acute cellular rejection; EBV, Epstein-Barr virus; MMF, mycophenolate mofetil; PTLD, post-transplant lymphoproliferative disorder; TL, telomere length.

## Abbreviations:

IPF: idiopathic pulmonary fibrosis
LTR: lung transplant recipient
TL: telomere length
MMF: mycophenolate mofetil
ACR: acute cellular rejection
PTLD: post-transplant lymphoproliferative disorder
EBV: epstein bar virus
CMV: cytomegalovirus
